# Clinical analysis of second-trimester pregnancy termination after previous caesarean delivery in 51 patients with placenta previa and placenta accreta spectrum: a retrospective study

**DOI:** 10.1186/s12884-021-04017-8

**Published:** 2021-08-18

**Authors:** Qiaofei Hu, Changdong Li, Lanrong Luo, Jian Li, Xiaofeng Zhang, Suwen Chen, Xiaokui Yang

**Affiliations:** 1 Department of Reproduction Regulation, Beijing Obstetrics and Gynecology Hospital, Capital Medical University, Beijing, 100026 China; 2 Department of Radiology, Beijing Obstetrics and Gynecology Hospital, Capital Medical University, Beijing, 100026 China; 3 Department of Human Reproductive Medicine, Beijing Obstetrics and Gynecology Hospital, Capital Medical University, Beijing, 100026 China

**Keywords:** Second-trimester pregnancy termination, Placenta previa, Placenta accreta, Management strategy

## Abstract

**Backgrounds:**

Pregnancy termination during the second trimester in patients with placenta previa and placenta accreta spectrum (PAS) is a complex and challenging clinical problem. Based on our literature review, there has been a relative increase in the number of such cases being treated by hysterotomy and/or local uterine lesion resection and repair. In the present study, a retrospective analysis was conducted to compare the clinical outcomes when different management strategies were used to terminate pregnancy in the patients with placenta previa and PAS.

**Methods:**

A total of 51 patients who underwent pregnancy termination in the second trimester in Beijing Obstetrics and Gynecology Hospital between June 2013 and December 2018 were retrospectively analyzed in this study. All patients having previous caesarean delivery (CD) were diagnosed with placenta previa status and PAS.

**Results:**

① Among the 51 patients, 16 cases received mifepristone and misoprostol medical termination, 15 cases received mifepristone and Rivanol medical termination, but 1 of them was transferred to hysterotomy due to failed labor induction, another 20 cases were performed planned hysterotomy. There was no placenta percreta cases and uterine artery embolization (UAE) was all performed before surgery.② There were 31 cases who underwent medical termination and 30 cases were vaginal delivery. Dilation and evacuation (D&E) were used in 20 cases of medical abortion failure and in all 30 cases of difficult manual removal of placental tissue. ③ A statistically significant difference was found among the three different strategies in terms of gestational weeks, the type of placenta previa status, main operative success rate and β-HCG regression time (*P* < 0.05). ④ There were 4(7.8%) cases who were taken up for hysterectomy because of life-threatening bleeding or severe bacteremia during or after delivery and hysterotomy. The uterus was preserved with the implanted placenta partly or completely left in situ in 47(92.2%) cases. Combined medical and/or surgical management were used for the residual placenta and the time of menstrual recovery was 52(range: 33 to 86) days after pregnancy termination.

**Conclusions:**

Terminating a pregnancy by vaginal delivery through medical induction of labor may be feasible if clinicians have an overall understanding of gestational age, the type of placenta previa status, the type of placenta accreta, and patients concerns about preserving fertility. A collaborative team effort in tertiary medical centers with a very experience MDT and combined application of multiple methods is required to optimize patient outcomes.

## Background

Caesarean scar pregnancy (CSP) is a rare ectopic pregnancy where the conceptus is implanted on the fibrous tissue of a previous cesarean scar defect [1, 2]. However, the incidence of CSP is increasing as the rate of cesarean delivery (CD) increases, and in response to the second-child policy in China [3]. The majority of CSP patients promptly terminate the pregnancy in the first trimester. Occasionally, CSPs progress to the second or third trimester and develop into placenta previa and placenta accreta spectrum (PAS) which may cause a life-threatening condition because of the high risk of uncontrolled hemorrhage, disseminated intravascular coagulation, uterine rupture, hysterectomy, and even death [4–6]. Terminating pregnancy in the second trimester for pregnant women with placenta previa and PAS and who have had a previous CD is both controversial and rarely reported. Currently, there is expert consensus in China on the preferred therapeutic protocol to guide the clinical diagnosis and treatment for these patients. However, there are still difficult challenges in the clinical management of these complex cases and few studies have reported on such cases, especially regarding vaginal delivery.

In the present study, we analyzed the clinical data of 51 patients with PAS in the presence of placenta previa status. All patients with a history of prior CD had undergone termination of second-trimester pregnancy in our hospital (reason for termination varied by patient). We analyzed the effectiveness of strategies for pregnancy termination and explored the feasibility of vaginal delivery in order to provide references for clinicians to minimize maternal injury, precisely evaluate the severity of these cases, and adopt the optimal individualized strategy.

## Method

### Patients

We retrospectively analyzed the clinical data of 51 inpatients treated in the Department of Reproduction Regulation, Beijing Obstetrics and Gynecology Hospital, Capital Medical University between June 2013 and December 2018. These patients presented with a singleton pregnancy diagnosed between 13 and 23 weeks of gestation and with placenta previa status and PAS and had a history of previous CD. They requested termination of pregnancy for different reasons such as severe fetal malformations, intrauterine fetal death, premature rupture of membranes, repeated vaginal bleeding before birth, or social factors. All procedures and management strategies were approved by the Human Ethics Committees of Beijing Obstetrics and Gynecology Hospital, Capital Medical University. Placenta previa status was defined as a placenta located in the lower uterine segment, the lower edge of the placenta approaching or reaching the internal cervical os, or partially or completely covering it (identified by ultrasonography in the mid-trimester) [7, 8]. The 2018 FIGO classification was used for the clinical diagnosis of PAS disorders, which included both adherent (creta) and invasive (increta and precreta) placenta disorders [9, 10].

### Inclusion and exclusion criteria

The inclusion criteria included: ①having a history of prior CD; ②total ultrasonography and partial magnetic resonance imaging (MRI) revealing second trimester pregnancy with placenta previa status and suspected prenatal diagnosis of PAS; and ③ PAS diagnosis having been confirmed by surgical procedure, postpartum clinical manifestations, imaging evidence or pathological examination [9–14]. The exclusion criteria were: ①receiving methotrexate (MTX) treatment or curettage before hospitalization; ②having contraindications for mifepristone, misoprostol, carboprost methylate or Rivanol; and ③experiencing functional failure of vital organs, aneurysm in the intubation pathway, a tendency to bleed persistently, or allergy to contrast agents.

### Management strategy

The management strategies included ① medical abortion with mifepristone (Zizhu Pharmaceutical Co., Beijing, China) and misoprostol (Zizhu Pharmaceutical Co., Beijing, China) (50 mg mifepristone orally, 2 times a day, for 2 days, 200 mg total, 400 μg misoprostol orally on the morning of the third day, repeat administration of 200–400 μg misoprostol at 3 h intervals, no more than 3 times) and uterine artery embolization (UAE) on the second day; **②** medical abortion with mifepristone and Rivanol (Hefeng Pharmaceutical Co., Donglan, Guangxi, China) (100 mg mifepristone orally, 2 times a day, 200 mg total, 100 mg Rivanol was injected into the amniotic cavity on the second day) and UAE on the second day before the injection of Rivanol; and③ planned hysterotomy within 24 h after UAE.

Dilation and evacuation (D&E) were used in some cases of medical abortion failure and in all medical abortion cases of difficult manual removal of placental tissue. In those cases who had heavy amounts of postpartum hemorrhage during delivery or hysterotomy (such as 5 min postpartum bleeding volume ≥ 80 mL or 30 min postpartum bleeding volume ≥ 300 mL), uterine balloon tamponade or intrauterine gauze packing for hemostasis at the site of placental insertion in the lower uterine segment was performed. Once uncontrollable bleeding occurred, hysterectomy was performed immediately.

### Follow-up

We applied perioperative antibiotics for all cases to prevent infection after pregnancy termination, and observed postoperative uterine contractions, vaginal bleeding, dorsal foot arterial pulse and skin temperature. The initial follow-up schedule was once per week after discharge to detect serum beta human chorionic gonadotropin (β-HCG). Serial sonographic examinations were performed and menstrual resurgence time were recorded. All patients (except for those who had hysterectomy) were advised to use contraception for at least six months.

### Statistical analysis

All data were analyzed using SPSS 21.0 software (IBM Corp., Armonk, NY, USA). The results are expressed as the means ± standard deviations (SDs), and normally distributed data were compared by means of one-way analysis of variance (ANOVA). Non-normally distributed data were compared using Kruskal–Wallis test. Bonferroni adjustment was performed for multiple comparisons. The chi-square test was used to compare rates among the groups. *P* values < 0.05 were considered to be statistically significant.

### Results

**Fig. 1:**
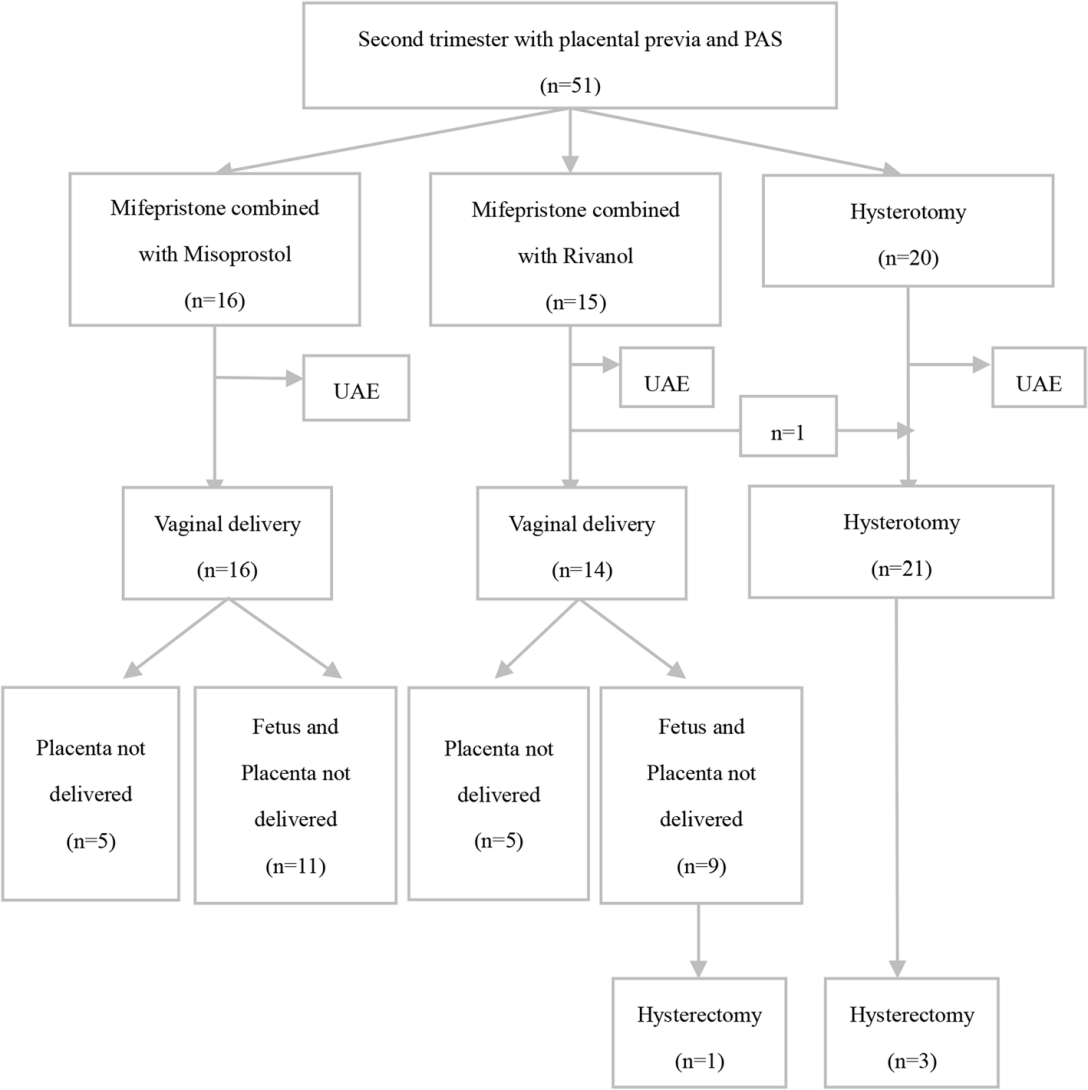
Therapy strategies and clinical outcomes of patients with placental previa and PAS in second trimester pregnancy

A total of 51 cases were included in this analysis. Sixteen cases received mifepristone and misoprostol medical abortion, 15 cases received mifepristone and Rivanol medical abortion but one of them was transferred to hysterotomy due to failed labor induction, another 20 cases were performed planned hysterotomy. There was no placenta percreta cases in our analysis and UAE was all performed before surgery. The mean age of the patients was 31.5 ± 4.4 (24–41) years, gestational age was 17.0 ± 2.9 (13–23) weeks, and gravidity and parity were 3.2 ± 1.1 (1–6) and 1.5 ± 1.1 (1–2), respectively. Therapy strategies and clinical outcomes of patients with placental previa and PAS in second trimester pregnancy are shown in Fig. 1.

**Fig. 2:**
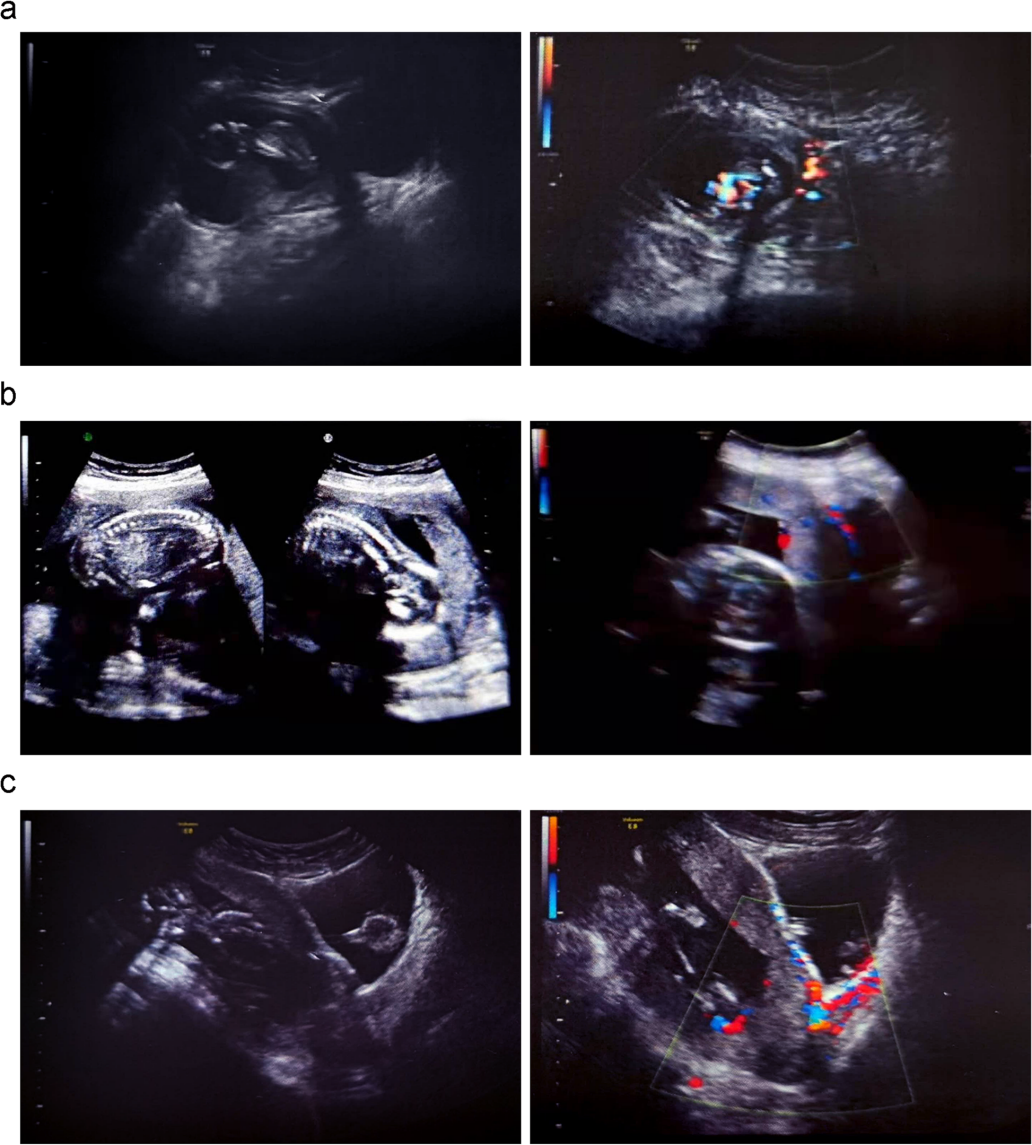
**a** Medical abortion with mifepristone and misoprostol at 13 weeks, **b** Medical abortion with mifepristone and Rivanol at 16 weeks, **c** Hysterotomy at 19 weeks

There were 31 cases who underwent medical abortion and 30 cases were vaginal delivery. Out of 30 cases, 20 cases with failed labor induction were terminated by D&E, and D&E were uesd in all 30 cases due to difficult manual or piecemeal removal of placental tissue. The bleeding volume of vaginal delivery cases was 605(range: 100 to 2000) ml in 24 h. The preoperative ultrasound of the three management strategies cases are shown in Fig. 2.

There were no statistically significant differences (*P* > 0.05) in demographic and clinical characteristics (such as age, gravidity, parity, years since last CD, clinical symptoms of the patients, type of PAS and thinnest muscle thickness of lower uterus) among the three different methods of pregnancy termination. But there were significant differences in gestational age and type of placental previa status (*P* < 0.05), as shown in Table 1. The number of complete placenta previa status was significantly more and the gestational age was significantly greater in the cases of hysterotomy (Table 1).

**Table 1:** Comparison of the patients’ demographic and clinical characteristics treated by the three different strategies (*n* = 51)

Items	Mifepristone + misoprostol	Mifepristone + Rivanol	Hysterotomy	p
	(*n* = 16)	(*n* = 14)	(*n* = 21)	
Age(y)				0.142
≤ 30	9(56.3)	9(64.3)	6(28.6)	
31–40	7(43.7)	5(35.7)	13(61.9)	
> 40	0	0	2(9.5)	
Gestational weeks(w)				0.000
13–16	16(100.0)	3(21.4)	4(19.1)	
17–20	0	8(57.2)	12(57.1)	
21–23	0	3(21.4)	5(23.8)	
Gravidity(n)				0.579
≤ 3	5(31.3)	3(21.4)	10(47.6)	
4–5	4(25.0)	4(28.6)	3(14.3)	
> 6	7(43.7)	7(50.0)	8(38.1)	
Parity(n)				0.148
1	9(56.3)	10(71.4)	8(38.1)	
2	7(43.7)	4(28.6)	13(61.9)	
Cesarean sections(n)				0.421
1	10(62.5)	10(71.4)	10(47.6)	
2	6(37.5)	4(28.6)	11(52.4)	
Years since the last cesarean sections(y)				0.325
≤ 2	5(31.3)	4(28.6)	2(9.5)	
3–5	8(50.0)	8(57.1)	11(52.4)	
> 5	3(18.7)	2(14.3)	8(38.1)	
Symptoms				0.982
None	11(55.0)	10(62.5)	13(48.1)	
Vaginal bleeding	4(20.0)	2(12.5)	6(22.2)	
Abdominal pain	3(15.0)	3(18.7)	5(18.5)	
Both	2(10.0)	1(6.3)	3(11.1)	
Thinnest thickness of lower uterus(mm)				0.063
≤ 0.5	4(25.0)	4(28.6)	9(42.9)	
0.5–3	4(25.0)	1(7.1)	8(38.1)	
> 3	8(50.0)	9(64.3)	4(19.0)	
Type of placenta previa status				0.013
Marginal type	5(31.3)	4(28.6)	3 (14.3)	
Partial type	5(31.3)*	1 (7.1)	0(0)*	
Complete type	6(37.5)*	9 (64.3)	18(85.7)*	
Type of PAS				0.403
Placenta Adherenta/creta	7(43.8)	7(50.0)	6(28.6)	
Placenta increta	9(56.3)	7(50.0)	15(71.4)	

Data are presented as count (%). χ^2^ test. *Bonferroni adjustment *p* < 0.017

No statistically significant differences were found in bleeding volume during the 24 h of surgery, rescue rate, blood transfusion rate, hysterectomy rate, length of hospital stay, serum β-HCG decline on the 3rd day after treatment, or menstrual recovery time (*P* > 0.05). There were significant statistical differences in β-HCG recovery time and main operative success rates among three different treatment strategies (*P* < 0.05) (Table 2).

**Table 2:** Comparison of patients’ intraoperative and postoperative conditions treated by the three different strategies (*n* = 51)

items	mifepristone + misoprostol	mifepristone + Rivanol	hysterotomy	p
	(*n* = 16)	(*n* = 14)	(*n* = 21)	
Bleeding volume in 24 h of surgery(mL)	672.50 ± 673.42	528.57 ± 610.08	752.38 ± 1310.58	0.626^b^
Rescue rate (n,%)	11(68.8)	8(57.1)	8(38.1)	0.168^c^
Blood transfusion rate (n,%)	6(37.5)	2(14.3)	4(19.0)	0.268^c^
Hysterectomy rate (n, %)	0(0)	1(7.1)	3(14.3)	0.286^c^
Main operative success rate (n,%)	5(31.3)	5(35.7)	18(85.7)	0.001^c^
Hospital stay(d)	10.69 ± 4.38	12.79 ± 8.40	11.57 ± 3.11	0.795^b^
Blood β-hCG decline of 3rd days after treatment (%)	92.79 ± 8.81	91.96 ± 8.33	91.85 ± 6.55	0.354^b^
Time of β-hCG recovery (d)	40.06 ± 1.56*	37.21 ± 2.39	32.95 ± 1.79*	0.028^a^
Time of menstrual recovery (d)	54.13 ± 2.67	55.50 ± 5.05	48.24 ± 4.03	0.195^b^
Residual placenta				0.739^c^
Expecting treatment	3(18.8)	4(28.6)	3(15.0)	
Medical treatment	10(62.5)	6(42.9)	10(50.0)	
Surgical treatment	3(18.8)	4(28.6)	7(35.0)	

Data are presented as mean ± SD and count (%)

^a^One-way analysis of variance, ^b^rank sum test of multiple independent samples, ^c^ χ^2^ test. *Bonferroni adjustment *p* < 0.017

**Fig. 3:**
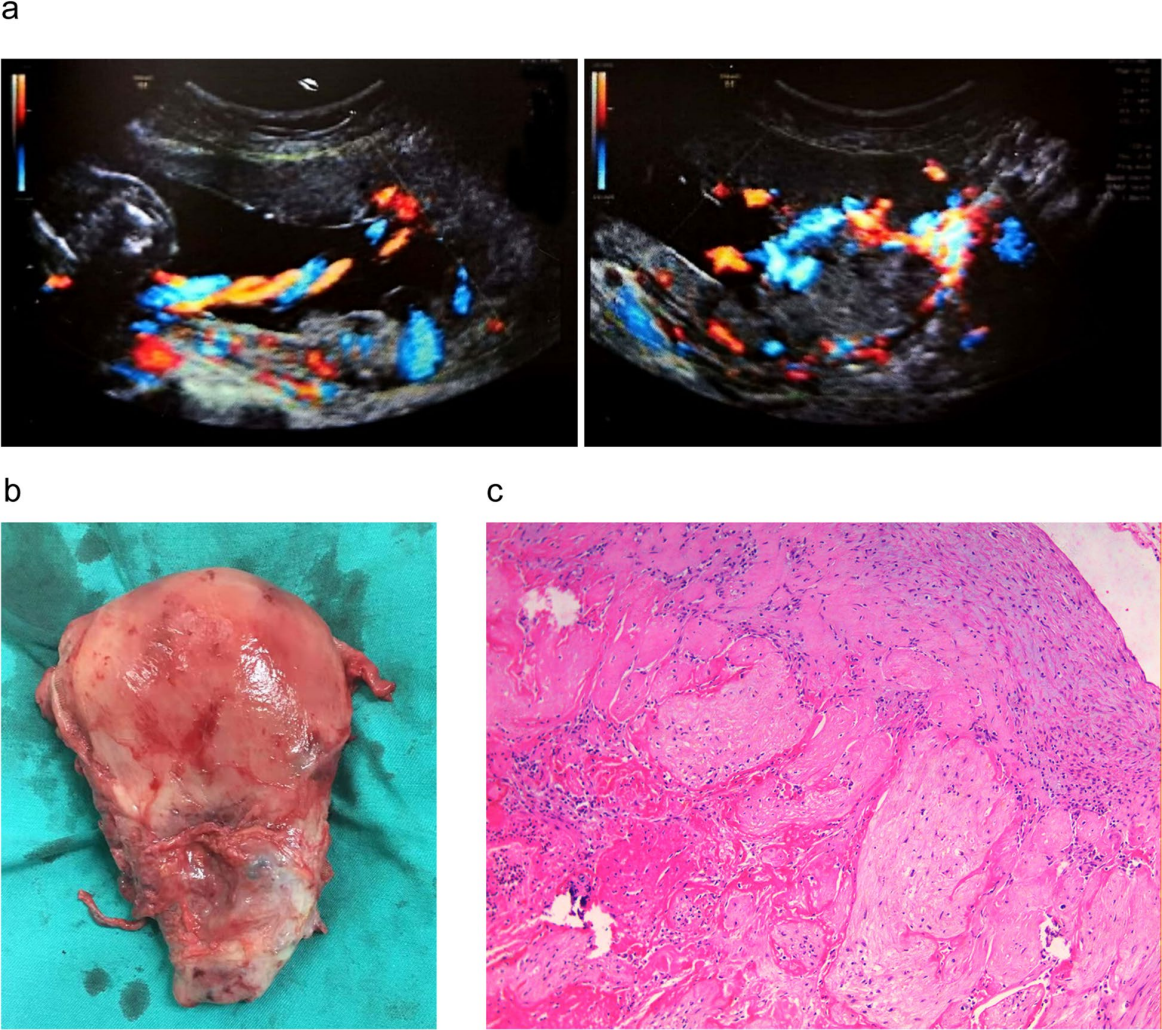
**a** Ultrasound before termination of pregnancy, **b** Hysterectomy specimen, **c** Pathology with 100x

**Fig. 4:**
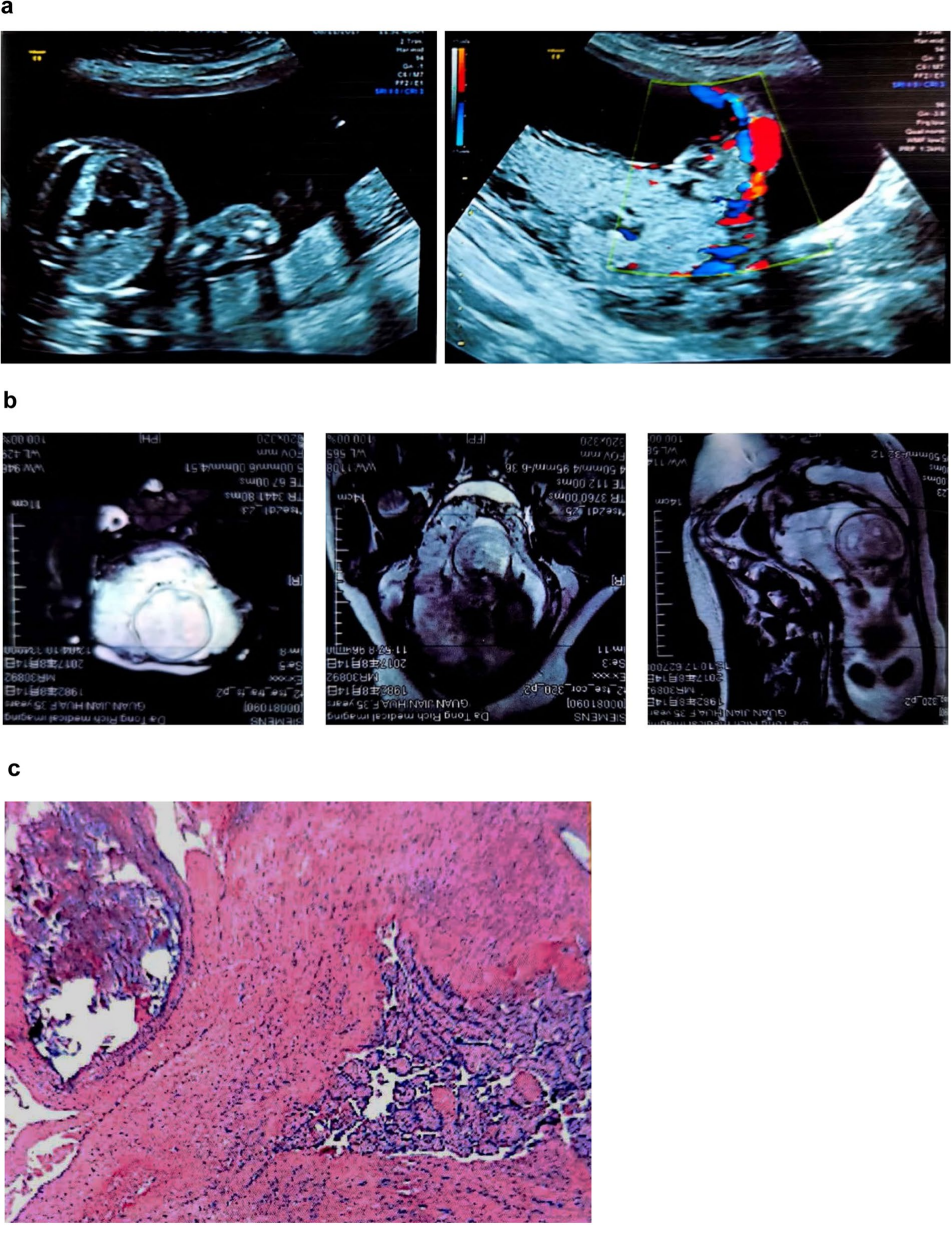
**a** Ultrasound before termination of pregnancy, **b** MRI before termination of pregnancy, **c** Pathology with 100x

Four cases (7.8%) performed hysterectomy during or after pregnancy termination. One case required hysterectomy to be performed during hysterotomy because of life-threatening bleeding and a poor response to multiple hemostatic measures. The remaining three cases were all related to residual placenta. One case was taken up hysterectomy because of secondary infection and intermittent fever after hysterotomy (Fig. 3). The last two cases were due to uncontrollable bleeding after hysterotomy (Fig. 4) and severe bacteremia after vaginal delivery. Another 47(92.2%) cases successfully retained the uterus with the implanted placenta partly or completely left in situ. Combined medical and/or surgical management were used for the residual placenta and the time of menstrual recovery was 52(range: 33 to 86) days after pregnancy termination.

## Discussion

The increase in cesarean section rates in the past two decades has led to an increase in the incidence of placenta previa and both adherent and invasive placenta accreta [15–17]. Patients with placenta previa status and PAS requiring an induced abortion on second-trimeste present a high risk of massive hemorrhage both during delivery and operation. There are few reports on these cases, so it is still unclear which treatment is most appropriate. An effectively tailored surgical approach is urgently needed to account for the serious life-threatening complications. In our study, the abortion success rate was high and the β-HCG regression time was short in the hysterotomy cases. Hysterotomy is relatively safe and effective for most patients who are more than 16 weeks of pregnancy with complete placental previa status and increta placenta. However, vaginal delivery by medically induced labor was feasible and the preferred method of management for some patients based on considerations such as gestational age, abnormal placental invasion, thickness of the lower uterine segment and communication with patients.

A report based on a meta-analysis indicated that the risk of placental previa increases as the number of prior CD increases, and women were at the highest risk of placenta accreta if they presented a low placenta or placenta previa in the second trimester of pregnancy with a previous history of CD [18]. In most middle- and high-income countries, the increase in the prevalence of PAS disorders is directly related to the increase in the cesarean section(CS) rate, as supported by epidemiological data [19, 20]. Second-trimester termination of pregnancy can pose potentially dangerous health concerns for these patients. In our study, the muscle layer of the lower segment of the anterior uterine wall was thinner, and the number of cases with complete placenta previa status was more and gestational weeks were greater in hysterotomy cases than the medical abortion cases (Table 1). Therefore, it is essential to fully evaluate gestational weeks, invasive placenta penetration, and abnormal vascular filling before planning individual treatment and procedures (Fig. 1). We recommended hysterotomy was preferred in patients with gestational age more than 16 weeks, complete placental previa status, and myometrial thinning to less than 0.5 mm or a large accreta area.

Matsuzaki et al. reported that the incidence of severe hemorrhage was high in patients with placenta previa and accreta with prior CD, whether vaginal delivery or CS was performed [21]. Given these multiple risk factors, it is rather difficult for a clinician to predict the severity of intraoperative bleeding prior to abortion. The mean blood loss was 644 mL (150–6000 mL) in our study. The 47(92.2%) cases successfully retained the uterus without severe complications or maternal death. We found no significant differences in operative blood loss and transfusion rate among the three different strategies which may be attributed to the small cases involved in our study (Table 2). Prophylactic bilateral UAE was performed for all patients to minimize heavy bleeding, as it is considered a safe and effective method of reducing the risk of morbidity or mortality. During treatment, we also used an intrauterine balloon tamponade or intrauterine gauze packing for hemostasis, however, intrauterine balloon tamponade can be served as a more rapid, effective method of controlling bleeding in patients who do not respond to medical treatment or suture ligation of the placenta site. The combined use of multiple modalities and multidisciplinary collaboration in our tertiary medical centers are crucial for the treatment of these patients.

As shown in Table 2, there was no significant difference in β-HCG decreases on the 3rd day after termination among the three different strategies, but the β-HCG regression time was significantly fast in the hysterotomy cases. Serum β-HCG recovery times ranged from 21 to 53 days. By analyzing the decrease in blood β-HCG, we can further determine the prognosis of patients, identify abnormalities in a timely manner, and administer corresponding treatments as quickly as possible.

Implementing vaginal delivery for second-trimester termination of pregnancy with placental previa and PAS is challenging, especially when future fertility preservation is a significant concern. There are also a few reports of inducing labor in such patients, which all suggested that patients with placental previa and PAS undergoing D&E in the second trimester had a high risk for hemorrhage, and prophylactic UAE and subsequent hysterectomy may be needed if hemorrhage occurs [21–25]. In our study, 31 cases underwent medical abortion at 13–23 weeks gestation, and 20 cases required D&E surgery for failed fetal abortion. All the 30 cases performed D&E due to difficult manual or piecemeal removal of placental tissue. In medical abortion, drugs played a role in softening and dilating the cervix, promoting fetal delivery, however, D&E was important and essential, especially for adherent or implanted placental tissue. Once patients suffered from severe bleeding or failed fetal abortion in the process of medical induction, hysterotomy or D&E was needed to terminate the pregnancy immediately.

Among the medical abortion cases, one case underwent hysterectomy due to secondary bacteremia that occurred three weeks after pregnancy termination. The case was 34 years old, gravida 6 para1, admitted at 23 weeks of pregnancy with complete placenta previa and placenta increta, no history of vaginal bleeding, and pain in abdomen. We thought that the infection was likely related to a prolonged period of medical abortion, the large area of placenta implantation, and the relatively long time of D&E procedure (45 min) after failed labor induction. Studies have also suggested that infection is one of the main reasons for hysterectomy after termination of pregnancy for this type of patient [26]. In the later period, we preformed hysterotomy for this similar cases and achieved good results.

The bleeding volume of vaginal delivery in 24 h was 605(range: 100 to 2000) ml. Given the high risk of hemorrhage and failed abortion, we should fully communicate the risks, benefits and alternative treatment options with the patients before abortion. At present, we rarely use Rivanol for induced labor because the induction time is uncontrollable and the success rate of abortion is low, unless those patients insist on maintaining the integrity of the uterus with marginal placenta previa status and adherent accreta. Therefore, we can infer that patients in the second trimester who have gestational age less than 16 weeks, marginal or partial placenta previa status, and adherent accreta or small area of increta can be prioritized for medical induced abortion.

For these cases, there is a tight connection between the placenta and the uterus, so the placenta can’t effectively be removed in its entirety and part of the placenta tissue can be purposefully left in situ to prevent uncontrollable bleeding. So, the management of residual placenta after induced labor or hysterotomy is another difficult problem for clinicians. Combined medical and surgical management including systemic MTX, UAE, curettage, hysteroscopic resection, or laparoscopic excision are needed in these cases [14, 27–29]. Studies have suggested that the overall success rate of uterus preservation in expectation therapy with residual placenta in situ is 78%, and the incidence of severe obstetric complications is 6% [30]. In our study, there were 10 cases who had heterogeneous echo < 1-2 cm by ultrasound and a significant decline in β-HCG without any additional treatment (Table 2). In addition, 26 cases with heterogeneous echo < 2–3 cm in the lower uterine segment and a slow decline in β-HCG were treated with medication, such as MTX, mifepristone and traditional Chinese medicine. Ultrasound-guided curettage or hysteroscopic removal of retained gestational products was performed in another 14 cases with heterogeneous echoes ≥ 3–5 cm or unsatisfactory decline in β-HCG. We preferred hysteroscopy to ultrasound-guided curettage, because the former had short operative time and direct visualisation for safe removal of retained gestational products.

After hysterotomy, two cases with residual placenta had to undergo hysterectomy. In one case, partial placental tissue left in situ, leading to a secondary infection and intermittent fever (Fig. 3). Therefore, hysterectomy was performed 28 days after the first surgery, even though the blood β-hCG was undetectable at that time. In the other case, residual placental tissue caused intermittent uncontrollable vaginal hemorrhage, which occurred 3^+^ months after surgery (Fig. 4). Therefore, we recommend that clinicians may consider simultaneous local uterine lesion resection and repair at the implantation site during hysterotomy to prevent the possibility of infection, delayed hemorrhaging, secondary hysterectomy, and potential complications if the residual tissue is large.

## Conclusions

Effective care of patients with concurrent placental previa status and PAS after prior CD who undergo second-trimester pregnancy termination requires a close collaborative team effort and thoughtful planning to ensure a good outcome. Although the small sample size prevents making a broad conclusion, the results suggest that vaginal medical induction of labor was a feasible treatment protocol for some patients in tertiary centers with a very experience MDT. Multi-center research is needed to explore the effects of different management strategies on the subsequent fertility of patients.

## Data Availability

The data used and analyzed during the current study are available from the authors on reasonable request.

## Abbreviations

PAS: Placenta accreta spectrum
CS: Cesarean section
CSP: Caesarean scar pregnancy
CD: Cesarean delivery
D&E: Dilatation and evacuation
MDT: Multi-Disciplinary Treatment
FIGO: International Federation of Gynecology
UAE: Uterine artery embolization
β-HCG: Beta human chorionic gonadotropin
