# Correction to: Clinical analysis of second-trimester pregnancy termination after previous caesarean delivery in 51 patients with placenta previa and placenta accreta spectrum: a retrospective study

**DOI:** 10.1186/s12884-021-04159-9

**Published:** 2021-10-09

**Authors:** Qiaofei Hu, Changdong Li, Lanrong Luo, Jian Li, Xiaofeng Zhang, Suwen Chen, Xiaokui Yang

**Affiliations:** 1 Department of Reproduction Regulation, Beijing Obstetrics and Gynecology Hospital, Capital Medical University, 100026 Beijing, China; 2 Department of Radiology, Beijing Obstetrics and Gynecology Hospital, Capital Medical University, 100026 Beijing, China; 3 Department of Human Reproductive Medicine, Beijing Obstetrics and Gynecology Hospital, Capital Medical University, 100026 Beijing, China

**Correction to: BMC Pregnancy Childbirth 21, 568 (2021)**

**https://doi.org/10.1186/s12884-021-04017-8**

Following publication of the original article [1], the authors reported an error in correspondence field of the PDF file. Corresponding affiliation in the bottom left corner of the first page for author Xiaokui Yang should be ‘Department of Human Reproductive Medicine, Beijing Obstetrics and Gynecology Hospital, Capital Medical University, Beijing 100,026, China’.

The original article [1] has been updated.
